# The microRNA repertoire of Tibetan naked carp *Gymnocypris przewalskii*: A case study in Schizothoracinae fish on the Tibetan Plateau

**DOI:** 10.1371/journal.pone.0174534

**Published:** 2017-03-30

**Authors:** Chao Tong, Fei Tian, Cunfang Zhang, Kai Zhao

**Affiliations:** 1 Key Laboratory of Adaptation and Evolution of Plateau Biota, Northwest Institute of Plateau Biology, Chinese Academy of Sciences, Xining, China; 2 Laboratory of Plateau Fish Evolutionary and Functional Genomics, Northwest Institute of Plateau Biology, Chinese Academy of Sciences, Xining, China; 3 Qinghai Key Laboratory of Animal Ecological Genomics, Northwest Institute of Plateau Biology, Chinese Academy of Sciences, Xining, China; 4 University of Chinese Academy of Sciences, Beijing, China; Xiamen University, CHINA

## Abstract

Tibetan naked carp *Gymnocypris przewalskii* is an ideal model system to study highland adaptation of fish, because it evolved specific genetic and phenotypic characteristics to adapt to chronic cold and alkaline environments in Lake Qinghai. MicroRNAs (miRNAs) are small noncoding RNAs that regulating gene expression post-transcriptionally in a wide range of biological processes. In this study, we focus on the role of miRNAs in adaptation of *G*. *przewalskii* to extreme conditions in Lake Qinghai. We generate the first miRNAome of *G*. *przewalskii* in Schizothoracinae fish. Using several genomic resources, we inferred 341 conserved miRNAs belonged to 152 miRNA families and 43 novel miRNAs in *G*. *przewalskii*, and also identified 15 teleost-specific miRNAs. Using a large scale of conserved miRNAs, we constructed a high-confidence phylogenetic tree between teleost and mammals than mitochondria and nuclear genes. In addition, we found that several miRNA family (e.g. miR-10 and let-7) members highly expressed in *G*. *przewalskii*, which may function in multiple biological processes. Finally, we predicted a total of 34,258 miRNA targets genes. Conserved miRNAs target genes participating in signal transduction, cell differentiation and biosynthetic process, and showed signature of functional constraint. While novel miRNAs in a species displayed species-specific targets and involved in ion binding, transport and oxidoreductase activity, may affect the expression patterns of targets with signature of gene family expansion or positive selection under extreme environment. Taken together, this study demonstrated that miRNAs may involve into roles of adaptation of *G*. *przewalskii* to highland aquatic environment, and also provide insights into miRNA regulatory network in Schizothoracinae fish as a case study.

## Introduction

MicroRNAs (miRNAs) are a family of endogenous RNAs that regulated gene expression pattern at post-transcriptional level. The miRNAs are usually 22 nucleotides (nt) long with a seed region (2–8 nt) that binds to the 3’ untranslated region (UTR) of a targeted mRNA and repressed the protein synthesis (plant: mRNA degradation). The miRNA have diverse functions, including developmental, metabolic and physiological processes [1,2]. Recent studies reported that miRNAs as vital regulators involved into a variety of pivotal biological processes in fish [3–7].

Schizothoracinae fishes are the predominant fish fauna in the Tibetan Plateau (TP), which have more than 60 species widely distributed throughout TP altitudes [8,9]. These fish species have evolved specific genetic and phenotypic characteristics to adapt to extremely harsh aquatic environments, such as chronic cold, hypoxia, saline and alkaline aquatic environments [8,10,11]. Tibetan naked carp, *Gymnocypris przewalskii* (Teleostei: Cyprinidae) is one of the best-studied Schizothoracinae fish species in TP, making it an ideal model to study evolutionary biology. Many studies have revealed that *G*. *przewalskii* provides many key insights into genetic basis of speciation and adaptation [12–16]. Unlike other Schizothoracinae fishes, *G*. *przewalskii* survives in both its natural habitant Lake Qinghai (high saline up to 13‰, high alkaline up to pH 9.4) and connective rivers of freshwater environment in the spawning migration. Besides high PH aquatic environment, *G*. *przewalskii* had evolved to surmount chronic cold and hypoxia environment in Lake Qinghai [8,17,18]. With unique characteristics, *G*. *przewalskii* is considered as an exceptional model to investigate the genetic mechanisms of aquatic wildlife adaptation to extreme environments in the TP.

Recent studies have identified key genes contributing to speciation and adaptation of *G*. *przewalskii *[12,16,19], but little is known on role of miRNAs in the adaptation of *G*. *przewalskii* to extreme aquatic environment in Lake Qinghai. Past evidences revealed that miRNAs could contribute to speciation and environmental adaptation in fish species [7,20]. Therefore, we combine small RNA sequencing of *G*. *przewalskii* and multiple bioinformatics analyses to uncover function of miRNAs in high altitude adaptation.

## Materials and methods

### Fish sampling

**Fig 1:**
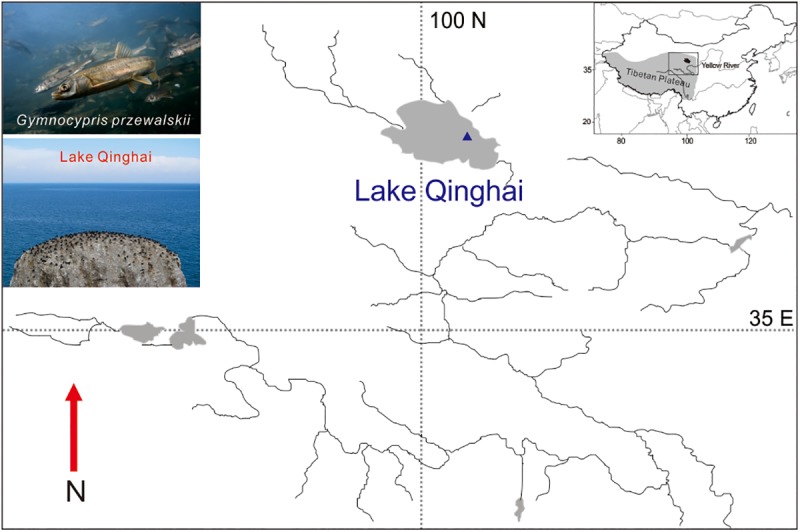
Sampling site and photos of *G*. *przewalskii* and Lake Qinghai. (a) The sampling map was created using the ArcGIS v10.1 (ESRI, CA, USA) and Adobe Illustrator CS5 (Adobe Systems Inc., San Francisco, CA). Blue triangle represents the sample site. Photos of *G*. *przewalskii* and Lake Qinghai were taken by Dr. Chao Tong in July 2015.

This study was based on wild-caught *G*. *przewalskii* that were collected in Lake Qinghai (37°03´N, 100°26´E, Fig 1), China, in July 2014 as adults and subsequently reared under laboratory conditions. Fishes experiments were approved by the Animal Care and Use Committees of the Northwest Institute of Plateau Biology, Chinese Academy of Sciences. The artificial insemination was based on a wild-caught female and a male of *G*. *przewalskii*. Fertilized eggs were collected and transferred to another re-circulating aerated freshwater system (12 h day/12 h night, 16°C). At 192 hour post fertilization (hpf), all the larvae of *G*. *przewalskii* were hatched out. Four *G*. *przewalskii* individuals (four replicates) were collected and then anaesthetized with MS-222 (2%, dipping treatment, Sigma, St. Louis, MO), finally frozen in liquid nitrogen prior to total RNA extraction for RNA library preparation followed by small RNA sequencing.

### Small RNA library construction for the Illumina sequencing

Total RNAs were isolated using TRIzol (Invitrogen, Carlsbad, CA) and then assessed the quality and quantity of each RNA sample with an Agilent 2100 bioanalyzer (Agilent Technologies, Palo Alto, CA). Only RNA samples with high purity (OD260/280 between 1.8 and 2.2) and high integrity (RNA integrity number, RIN value more than 7.0) were used to construct the small RNA library [21]. Approximately 20 μg of total RNA each was used with an Illumina TruSeq Small RNA Sample Prep Kit (Illumina, San Diego, California, USA) for small RNA transcriptome library preparation, and sequenced on an Illumina HiSeq™ 2500 instrument. The raw data of small RNA-seq have been submitted to the NCBI Sequence Read Archive database (https://www.ncbi.nlm.nih.gov/sra).

### Sequence analysis for miRNA identification

Small RNA-seq raw reads were treated by following procedures: (1) trimming contaminating (5’ adaptor contaminants, no insert tags, oversize insertions, poly (A) tags and small tags); (2) removing poor-quality reads and resulting in clean read (total sequence); (3) reads with same sequence were named unique sequence. Numbers and lengths of total and unique sequences were calculated, respectively. Then all the sequences were annotated by searching against NCBI Genbank noncoding (Nr) database (http://blast.ncbi.nlm.nih.gov/) and Rfam database (http://www.sanger.ac.uk/software/Rfam). The number and frequency of total and unique sequences of miRNA, tRNA, tRNA, rRNA, snoRNA and intro were also respectively calculated. Only the miRNAs in unique sequence were considered as candidates for miRNA annotation. Therefore, the putative miRNAs ranging from 15 to 30 nt were searched against known precursor/mature miRNAs in miRBase release 20 database [22] of selected 8 vertebrate species (*Danio rerio*, *Oryzias latipes*, *Tetraodon nigroviridis*, *Pan troglodytes*, *Bos taurus*, *Gallus gallus*, *Homo sapiens*, *Rattus norvegicus*) to identify conserved miRNAs. Considering the difference among species, alignments of the putative miRNAs to miRNA precursor/mature miRNA of above selected animal species in miRBase allowed for no more than three mismatches.

A draft genome assembly of *G*. *przewalskii* is not yet available, while a well annotated zebrafish genome could be used as a reference. The above remaining unannotated unique sequences were considered as putative novel miRNAs. Then MIREAP software (http://sourceforge.net/projects/mireap) was used to predict putative novel miRNA precursors against the zebrafish genome with the following criteria: (1) candidates are unannotated sequences which match the reference genome, aligning to intronic regions or to antisense exon regions; (2) sequences and structures (stem-loop structures) of the genes which satisfy the criteria of hairpin miRNA formation, and that the mature miRNAs are present in one arm of hairpin precursors; (3) mature miRNA strand and its complementary strand (miRNA*) should contain 2-nt 3’-overhangs; (4) hairpin precursors do not contain large internal loops or bulges; (5) secondary structures of hairpins have minimum free energy (MFE) less than −18 kcal/mol [23]; (6) the number of mature miRNAs with predicted hairpins should be more than 5 in alignment result. The miRNAs meeting all these criteria were considered as novel miRNAs in *G*. *przewalskii*. Finally, we analyze and compared the bias of each nucleotide position and the first nucleotide of above identified conserved and novel miRNAs in *G*. *przewalskii*.

### miRNA family identification and phylogenetic analysis

After aligning miRNAs and their precursors to above 8 animal species miRNAs deposited in miRBase using Clustal X and MEGA 5.0 [24,25], only sequences with higher than 98% homology and less than two mismatches were predicted to be potential miRNA family members from the conserved miRNA families in animals. Then cluster analysis was used to evaluate the conservation and diversity of identified miRNA families between *G*. *przewalskii* and other 8 animal species, and was finally illustrated by heatmap with ggplot2 package in R software [26].

All identified conserved miRNA families of *G*. *przewalskii* and other 8 animal species were treated as Dollo type characters and assigned equal weight [27]. Then, phylogenetic analyses were performed with branch-and-bound Maximum Parsimony (MP) search [28] implemented in PAUP software v.4.0b10 [29]. Bayesian analysis was performed using BEAST software v.1.8 [30] with parameter settings following Field *et al*. [31].

### miRNA expression profiling analysis

To evaluate and calculate miRNA abundance in each replicate libraries, small RNA reads were mapped into identified conserved miRNA of *G*. *przewalskii* according to previous method [32]. Firstly, the average count of each conserved miRNA was calculated respectively for further comparison. Secondly, the range of average expression levels of miRNAs was also calculated. Thirdly, highly expressed miRNAs were also identified and classified into different miRNA families.

### miRNA target gene prediction and functional annotation

*In silico* analysis of miRNA target was predicted in published mRNA resources [12,14] using miRanda 4.0 algorithm (microrna.sanger.ac.uk/targets/v4) and TargetScan (www.targetscan.org), based on the complementary region between miRNAs and mRNAs and the thermodynamic stability of miRNA-mRNA duplex. All the miRNA target mRNAs were calculated and clustered by Gene Ontology (GO) terms using Blast2GO software [33].

## Results

### Characterization of small RNAs in *G*. *przewalskii*

**Fig 2:**
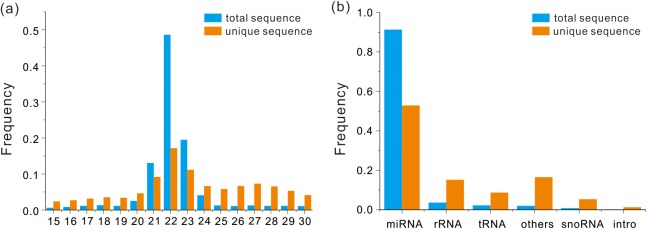
Comparison of total and unique sequence in *G*. *przewalskii*. (a) Size distribution and frequency of miRNAs in both types of sequences. The 22-nt-length of small RNA is abundant in both types. (b) Frequency of total and unique sequences of putative RNAs, including miRNA, tRNA, tRNA, rRNA, snoRNA and intro. The most abundant of RNAs are miRNA in both types of sequences. The total and unique sequences were colored by blue and orange, respectively.

Total 60,037,248 raw reads and 47,030,422 (78.34%) clean reads were generated by small RNA sequencing (S1 Table). Then clean reads from 4 libraries were merged for subsequent analysis. After length selection, 37,552,615 of total sequence were left ranging from 15 to 30 nucleotides (nt). A total of 564,579 of unique sequences were obtained by the cluster analysis. The number and length of total and unique sequences were respectively calculated, which showed that high percentage of clean reads was 22 nt long for total and unique sequences (Fig 2A and S2 Table). After annotation by Nr and Rfam databases, the miRNA, rRNA, tRNA, snRNA, snoRNA and other non-coding RNAs in total and unique sequences were identified. The most abundant of small RNAs are miRNA in both types of sequences (Fig 2B and S3 Table).

### Conserved and novel miRNAs in *G*. *przewalskii*

**Fig 3:**
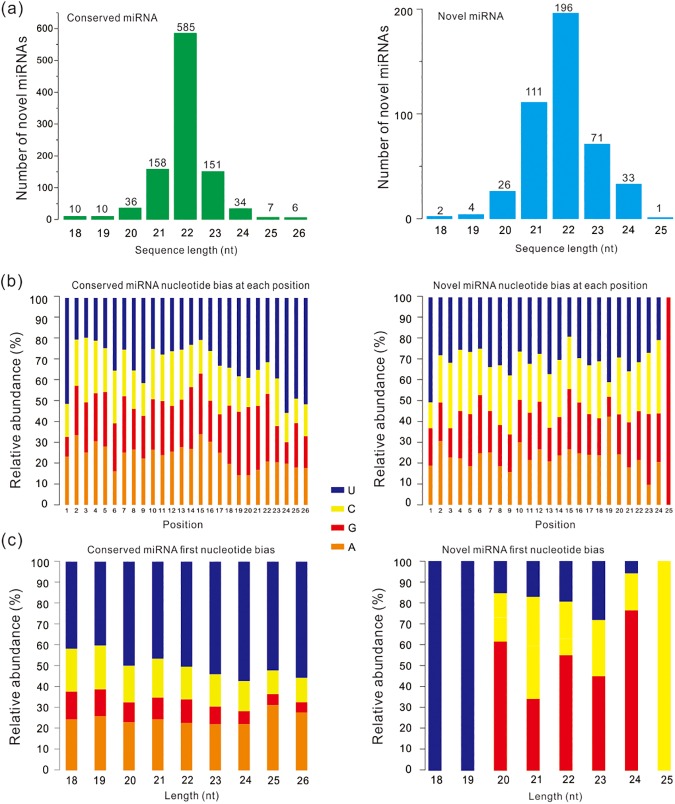
Size distribution and nucleotide bias of conserved and novel miRNAs in *G*. *przewalskii*. (a) Size distribution of conserved and novel miRNAs in *G*. *przewalskii*. As the percentage of miRNAs, the most abundant miRNAs were 22-nt long. (b) Relative abundances of conserved and novel miRNA nucleotide bias at each position. The base of conserved and novel miRNAs at each position was most biased toward U. (c) Relative abundances of first nucleotide bias in conserved and novel miRNAs. The conserved miRNAs were most biased toward U at the first-base position. While the scenario is complex in novel miRNAs, the first-base position is toward different base for different length of miRNAs.

We conducted sequence homology search of candidate miRNAs against known precursor/mature miRNAs in miRBase release 20 database [22], and identified 998 conserved miRNAs within *G*. *przewalskii* and other 8 animal species (S4 Table). Using the zebrafish genome as a reference, we identified 43 novel miRNAs in *G*. *przewalskii*, which met the above consensus criteria (see Materials and Methods, S5 Table). Then we compared the size distribution between conserved and novel miRNAs in *G*. *przewalskii* and found that 22-nt long miRNA is dominant in both categories (Fig 3A). In addition, as shown in Fig 3B, U (uracil) is the dominant biased base in each nucleotide position in both conserved (1-26nt) and novel miRNAs (1-25nt). Noteworthy, the miRNA length ranging from 20 to 24 nt with high frequency were taken into consideration for miRNA first nucleotide bias analysis, showing that majority of conserved miRNAs tended to start with 5’-U while the novel miRNA is biased towards G. Notably, no A was found in the first nucleotide of novel miRNAs in *G*. *przewalskii* (Fig 3C).

### Phylogeny inferred from miRNAome

**Fig 4:**
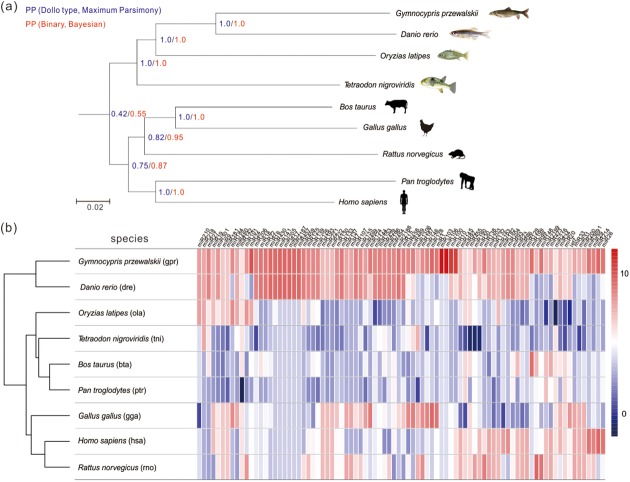
Phylogenetic position of *G*. *przewalskii* and clustering analysis result based on miRNA-wide scale datasets. (a) The miRNA data suggest that *G*. *przewalskii* is closely related to *D*. *rerio*, and in the branch of teleost fishes. Posterior probabilities (PP) of Maximum Parsimony (blue) and Bayesian (red) in each node are marked, respectfully. (b) Comparison of conserved miRNA families in *G*. *przewalskii* and other 8 animal species shown in heatmap plot. The miRNA family data of other vertebrates were downloaded from miRBase 21.0. Rows represent each species with abbreviated miRBase three-letter names parenthesized. Columns indicate the each miRNA families. Color coding was used to indicate the number of miRNA members, with dark red corresponding to the highest number (10) and dark blue the lowest (0).

Using alignment and cluster searches based on precursor and mature miRNAs, we identified 152 potential miRNA families in *G*. *przewalskii* from 341 members (S6 Table). A phylogenetic tree was constructed based on sequences of 152 miRNA families using both Maximum Parsimony and Bayesian methods (Fig 4A). All the teleost fishes and mammal species were clearly separated into two groups with high posterior probability (PP = 1 in teleost group, PP > 0.7 in mammal group). In consistent with the hierarchical clustering analysis result, *G*. *przewalskii* and *D*. *rerio* were closest in phylogenetic position.

### Conservation and diversity of predicted miRNA families in *G*. *przewalskii*

The largest miRNA families were miR30 and let-7, which both had ten members (Table 1). Notably, a majority (42.76%) of miRNA families had only one member, similar to other animal species. In addition, to comprehensively understand the conservation and diversity of miRNA families in different animal species, we further compared the number of each miRNA families within selected animal species by hierarchical clustering analysis. As shown in Fig 4B, the miRNA families in *G*. *przewalskii* and *D*. *rerio* were highly homologous. While the remaining two fish species, *O*. *latipes* and *T*. *nigroviridis* were clustered into a group with mammals, including *B*. *taurus* and *P*. *troglodytes*. Furthermore, we also found that 15 miRNA families were absent in mammals but only investigated in teleost fishes, such as miR-722, miR-729 and miR-731 (S6 Table).

**Table 1:** Number of member in each identified conserved miRNA family.

Size of family member	Number of miRNA family	Percent of conserved miRNA family (%)
1	63	41.45
2	45	29.61
3	6	3.95
4	20	13.15
5	6	3.94
6	5	3.28
7	2	1.31
8	2	1.31
9	1	0.06
10	2	1.31

### miRNA expression profiling and highly expressed miRNA family

Based on miRNA expression analysis result (S7 Table), it was clear that four replicate small RNA-seq libraries had a high correlation. After comparison of range of miRNA expression levels, we found that a majority of expressed miRNA with read counts ranged from 10 to 100 (n = 333). We also identified 85 highly expressed miRNAs with more than 10,000 read counts. To get a clearer perspective of miRNA expression levels, we had compared several miRNA families that have highest reads numbers in *G*. *przewalskii*. At first, we focused on the miR-10 family which contained 5 members in top 20 highly expressed miRNAs, including miR-10c-5p, miR-10d, miR-10a-5p, miR-10c, miR-10a-3p (S7 Table). Another highly expressed miRNA family is let-7, such as let-7a-5p, let-7g-5p, let-7i-5p, let-7d-5p, let-7e-5p (S7 Table). The third miRNA family is the tissue-specific miRNA, such as miR-722 and miR-731 (S7 Table).

### Target genes annotation of miRNAs in *G*. *przewalskii*

After bioinformatics analysis of miRNA and potential target mRNA retrieval from transcriptome data, we totally identified 6,315 genes as targets of 384 miRNAs, of which 4,936 and 1,379 targets were mediated by conserved and novel miRNA. Target genes were annotated by GO using Blast2GO software (S8 and S9 Tables). For the conserved miRNA targets, majority of target genes were enriched into signal transduction, cell differentiation and biosynthetic process (Table 2 and S8 Table). The novel miRNA targets mainly involved in ion binding, transport and oxidoreductase activity (Table 2 and S9 Table), such as solute carrier (SLC) family (SLC9A3 and SLC19A3) and transmembrane protein (TM) family (TM9, TM33, TM97, TM120, TM175) were involved in transport function. NDUB9, COX11, MDH, ATP5c1 and ATP5b were involved in ATP binding and oxidative phosphorylation (S9 Table).

**Table 2:** Number of conserved and novel miRNAs target genes in the top 5 ranked GO terms.

GO term	Number of genes
**conserved miRNAs**	
signal transduction	140
cell differentiation	129
oxidoreductase activity	123
small molecule metabolic process	102
response to stress	95
**novel miRNAs**	
ion binding	73
cellular nitrogen compound metabolic process	70
biosynthetic process	46
signal transduction	43
transport	40

## Discussion

The big progresses were made in unveiling mechanisms of speciation and environmental adaptation of *G*. *przewalskii*, especially the contributions from functional genes [10,12,13,16–18]. Besides functional genes, miRNAs had been acknowledged as powerful regulators in environmental adaptation and potential drivers of speciation [34–36]. Here we generated and annotated the first comprehensive small RNA transcriptome resource for *G*. *przewalskii*. This study would shed lights on the adaptation of *G*. *przewalskii* to extreme environment and explored roles of miRNAs in highland adaptation of fish.

We found high miRNA sequence similarity between *G*. *przewalskii* and other 9 animal species analyzed here, and identified a number of conserved miRNAs. It was well known that vertebrate miRNAs and their precursors were conserved among diverse animals [37]. The nucleotide bias in conserved miRNA was similar between *G*. *przewalskii* and other cyprinid fishes, including common carp, bighead carp and silver carp [32,38]. The nucleotide bias in novel miRNAs was varied among animal species [32,35,38,39], implied that functional divergence of non-conserved miRNAs was probably species-specific. In addition, we found that *G*. *przewalskii* shared similar number of conserved miRNA families with other animal species, especially teleost [32,38,39]. However, the number of members of miRNA families was varied, which was also confirmed by hierarchical clustering analysis (two teleost *O*. *latipes* and *T*. *nigroviridis* and two *B*. *taurus* and *P*. *troglodytes* were clustered into one group). We suggested that this may because the incomplete annotation of teleost genomes. Moreover, we identified 15 teleost-specific miRNAs, in consistent with recent studies [40,41]. For instance, miR-722 identified by our and others, was confirmed to function in immune system development and immune response [41]. These finding implied that teleost fish presumably had several different regulatory networks with mammals.

The miRNA showed higher level of conservation than protein-coding sequence, which had been confirmed by previous studies [20,31,40,42]. There are two important implications in this study. First, we obtained a phylogenetic tree of selected teleosts and mammals with relatively high supporting rate than previous phylogeny based on several mitochondria and nuclear makers [43]. Therefore this study showed a case for further phylogenomic analyses within Schizothoracinae fish based on miRNAome in the future. Second, conserved miRNA had been acknowledged as functional constraint acting on their target genes [2,37]. Another evidence was that purifying selection was acting on most of genes [44]. We could infer that genes regulated by conserved miRNAs may be also functional constraint under purifying selection. Therefore, it was not surprising that GO functional enrichment analysis of conserved miRNA targets showed similar results with other teleost fish [32,38,39].

In general, abundant miRNAs play fundamental and broader regulatory functions [32,40,45]. We did not identify many highest expressed miRNAs and only 85 miRNAs occurred (over 10,000 read counts). For instance, three members of miR-10 (miR-10c-5p, miR-10d and miR-5p) contained even more than 100,000 read counts. The members of miR-10 family dominate the sex determination and development of animal gonad [46]. The sequencing samples were all larval *G*. *przewalskii*, therefore, these highly expressed miR-10 family members may play important roles in sex differentiation at early stage. This finding was also consisted of previous study in development of tilapia gonads [4]. In addition, a number of let-7 family members were highly expressed in *G*. *przewalskii*. We also found that let-7 family had the largest number of family members in *G*. *przewalskii*. Past evidences revealed that let-7 have diverse functions involved into different biological processes, including cell proliferation, development, growth and immune [47]. These findings implied that highly expressed let-7 family members may also functional constraint and involved in multiple biological activities of *G*. *przewalskii*. Furthermore, we also identified a number of species-specific miRNAs highly expressed in *G*. *przewalskii*. To date, functions of many specific miRNAs are still not well-understood. Therefore, whether the functions of species-specific miRNAs are related to organism adaptation to specific environments, these issues remain as interesting questions for further confirm.

Most of the novel miRNAs in a species displayed species-specific targets [20,45]. In this study, functional enrichment (GO) analyses indicated that majority of novel miRNAs target genes involved in ion binding, transport and oxidoreductase activity. Recent evidences also revealed that genes related to ion binding, transport and energy metabolism showed signals of positive selection in *G*. *przewalskii* [12]. Another evidences reported that gene family of energy metabolism and hypoxia response had expanded in several Schizothoracinae fish species, such as *G*. *pachycheilus* and *G*. *przewalskii* [48,49]. The miRNA are known to regulated gene expression at posttranscriptional level [2]. As the fact of *G*. *przewalskii* inhabited Lake Qinghai in an extremely saline and alkaline, hypoxic and chronic cold environment, its gene regulatory network may adapt these different ecological niches during long-term evolution. We speculated that these novel miRNAs may affect the expression patterns of these genes with signature of gene family expansion or positive selection, and be potential drivers of ultimately speciation of *G*. *przewalskii*. However, this hypothesis needs to be further confirmed by comprehensive verification works in future.

In summary, this case study in *G*. *przewalskii* would provide insights for future integrated studies focusing on determining potential roles of miRNAs in different ecological niches adaptation and speciation in Schizothoracinae fish on the Tibetan Plateau.

## Supporting information

S1 Table Information of small RNA sequencing. (XLSX)

S2 Table Size distribution of small RNAs in both types of sequences. (XLSX)

S3 Table Information of annotated total and unique sequences by Nr and Rfam databases. (XLSX)

S4 Table Conserved miRNAs within 9 species. (XLSX)

S5 Table Information of novel miRNAs. (XLSX)

S6 Table Gene family number of conserved microRNAs within 9 species. (XLSX)

S7 Table Expression of conserved miRNAs in *G*. *przewalskii*. (XLSX)

S8 Table GO annotation of conserved miRNA targets. (XLSX)

S9 Table GO annotation of novel miRNA targets. (XLSX)
